# Combined systemic immune-inflammatory index and prognostic nutritional index predicts the efficacy and prognosis of ES-SCLC patients receiving PD-L1 inhibitors combined with first-line chemotherapy

**DOI:** 10.3389/fonc.2024.1485849

**Published:** 2024-12-04

**Authors:** Yi Ge, Xiaoyu Liu, Yishi Xu, Yanwei Su, Yixin Li, Liping Wang

**Affiliations:** ^1^ Department of Oncology, First Affiliated Hospital of Zhengzhou University, Zhengzhou, China; ^2^ Department of Oncology, Luohe Central Hospital, Luohe, China

**Keywords:** small cell lung cancer, PD-L1 inhibitors, systemic immune-inflammation index, prognostic nutritional index, survival

## Abstract

**Background:**

There is a strong association between inflammation and the formation, progression, and metastasis of malignant tumors, according to earlier studies. Some composite inflammation-nutritional indicators, such as the systemic immune-inflammation index (SII) and the prognostic nutritional index (PNI), have a certain predictive effect on the prognosis of patients with small cell lung cancer (SCLC). However, the relationship between these indicators and the efficacy of immunotherapy in SCLC patients is still not well understood. Therefore, the purpose of this study was to explore how the pre-treatment SII-PNI score can predict the tumor response and prognosis of extensive-stage SCLC patients treated with PD-L1 inhibitors and first-line chemotherapy.

**Methods:**

This research conducted a retrospective review of 70 ES- SCLC patients from December 2019 to January 2023. According to the SII-PNI score, all patients were categorized into three groups. Overall survival (OS) was assessed by implementing the Kaplan Meier and Cox regression models. In addition, we devised a nomogram and scrutinized its accuracy in prediction through receiver operating characteristic (ROC) curve analysis and visualized it by calibration plots. Subsequently, a risk classification system was established.

**Results:**

Patients with higher SII-PNI scores exhibited notably poorer survival outcomes compared to their counterpart with low SII-PNI score (p=0.008), as well as poorer short-term curative effects (p=0.004). The results of the multivariate analysis revealed that the SII-PNI score (p=0.036) had an independent association with a less favorable OS. The nomogram has been demonstrated to be a reliable prognostic tool for ES-SCLC patients. A notable difference was identified between the two different levels of risk.

**Conclusion:**

The baseline SII-PNI score can serve as a reliable prognostic indicator for ES-SCLC patients receiving immunotherapy. Higher SII-PNI scores imply a worse prognosis.

## 1 Introduction

Lung cancer stands as the most common reason for mortality from malignant tumors worldwide. It is typically classified into two main categories. SCLC constitutes approximately 15% of all lung cancers (1, 2). Compared with NSCLC, SCLC has the characteristics of higher malignancy, shorter tumor cell doubling time, and an earlier susceptibility to distant metastasis (3). A great deal of SCLC patients are already in the extensive stage when diagnosed, with extremely poor clinical prognosis (4). Over the past three decades, platinum combined with etoposide chemotherapy remains the typical treatment for SCLC (5). In the last several years, the use of PD-L1 inhibitors to treat SCLC patients has made tremendous progress. In the phase III clinical trial known as IMpower133, the combination of Atezolizumab with first-line chemotherapy resulted in an increased OS for patients compared to chemotherapy alone. In another clinical study CASPIAN, found that the median OS of patients receiving the combination of Durvalumab and chemotherapy was 2.7 months longer than the control group (6, 7). Durvalumab and Atezolizumab were approved as one of the first-line treatments for SCLC in February and March 2020, respectively.

However, immune checkpoint inhibitors (ICIs) can also lead to a range of immune-related adverse events (irAEs), including rash, pruritus, pneumonitis, diarrhea, and endocrine system problems (8). Only a portion of SCLC patients can benefit from immunotherapy, so it is crucial to search for biomarkers that can predict the prognosis and accurately identify subgroups of patients suitable for immunotherapy. At present, numerous studies have explored biomarkers for the efficacy of immunotherapy, proposing the predictive value of PD-L1 expression level, tumor mutation burden (TMB), tumor microenvironment, etc. (9). However, these biomarkers face difficulties in clinical application due to limitations in tissue sampling, inconsistent detection standards, and tumor expression heterogeneity. Their predictive value for the efficacy of SCLC immunotherapy is still unclear. Numerous investigations have revealed that the occurrence, development, invasion, metastasis, and anti-tumor response of tumors are strongly influenced by inflammation, immunity, and overall nutritional status (10). Research have found that some composite inflammatory indicators, such as the systemic immune-inflammation index (SII) and the prognostic nutritional index (PNI), can predict the prognosis of many malignant tumors, especially lung cancer (11–15). These hematological inflammatory biomarkers are inexpensive and readily accessible, but their predictive effectiveness in the efficacy of immunotherapy for ES-SCLC remains largely uncertain.

The SII-PNI is highly useful in predicting the curative effect and prognosis of non-small cell lung cancer (16) and gastric cancer (17) patients in earlier studies. The predictive ability of SII-PNI score for the efficacy and prognosis of ES-SCLC patients undergoing immunotherapy is the main focus of this study, providing guidance for screening the most suitable patients for immunotherapy.

## 2 Materials and methods

### 2.1 Participants and study design

We selected all 70 eligible ES-SCLC patients who received PD-L1 inhibitors combined with first-line chemotherapy when newly diagnosed from December 2019 to June 2022 at the First Affiliated Hospital of Zhengzhou University. The next selection standards were adopted: (1) small cell lung cancer diagnosed through histological or cytological examination; (2) ES-SCLC evaluated according to the VALSG staging criteria; (3) normal organ functionality to withstand immunotherapy and chemotherapy; (4) ECOG activity status score ≤ 2; (5) no other coexisting malignant tumors; (6) expected survival > 3 months; (7) there was at least one measurable lesion that can be assessed using response evaluation criteria in solid tumors (RECIST). The exclusive standards are as follows: (1) previously received systemic anti-tumor therapy; (2) unable to make a clear pathological diagnosis or unclear primary lesion; (3) active autoimmune diseases still require hormone therapy; (4) combined with other primary tumors; (5) suffering from severe lung and heart diseases, etc.; (6) ECOG score > 2. The study was conducted following the guidelines of Declaration of Helsinki and approved by the Ethics Committee of the First Affiliated Hospital of Zhengzhou University (2024-KY-1197-001).

### 2.2 Chemotherapy regimen

The administration regimen was as follows: (1) Atezolizumab 1200 mg, intravenous infusion on the first day (the first infusion should last for at least 60 minutes; if well tolerated, subsequent infusions should last for at least 30 minutes) or Durvalumab 1500 mg, intravenous infusion on the first day (infusion time 60 minutes). (2) etoposide injection 100mg/m², intravenous infusion for 1-3 days. (3) carboplatin injection AUC=5, intravenous infusion on the first day, or cisplatin injection 75mg/m², intravenous infusion on the first day. All included patients were treated with PD-L1 inhibitors in combination with chemotherapy with a cycle of 3 weeks. After 4-6 cycles of treatment, PD-L1 inhibitor monotherapy was maintained until disease progression occurred. Whether or not to combine radiotherapy is based on the patient’s condition.

### 2.3 Data collection and definition

Collect clinical pathological data and pre-immunotherapy hematological examination results, especially age, gender, Body Mass Index (BMI), ECOG scores, smoking history, extrapulmonary metastases; hematological indicators before receiving PD-L1 inhibitor treatment, including blood routine, liver and kidney function, C-reactive protein (CRP), and tumor markers, etc. Collect fasting peripheral venous blood samples within one week before immunotherapy. Inflammatory indicators were determined using the following formulas: (1) SII = platelet count(/L) × neutrophil count(/L)/lymphocyte count(/L). (2) PNI = serum albumin(g/L)/+5 × lymphocyte count (/L).

### 2.4 Follow up

All participants received consistent follow-up through a combination of telephone, inpatient medical records, and outpatient review, with a follow-up deadline of January 20, 2023. Imaging evaluations were as follows: electronic computed tomography (performed every 42 days), color Doppler flow imaging (performed every 42 days), cranial magnetic resonance imaging (performed every 3months), and whole-body bone scanning (performed every 6 months). Tumor response was evaluated every two cycles (42 days) through imaging examinations, tumor markers, symptoms, etc., using Response Evaluation Criteria in Solid Tumors (RECIST) 1.1 by clinical doctors. The patients’ reactions to treatment were classified as complete response (CR), partial response (PR), stable disease (SD), and progressive disease (PD). The definition of Partial response (PR): The total diameter of all measurable target lesions is≥30% lower than baseline. All target lesions must be evaluated. For conflicting assessments, we focused on the overall tumor burden and determined treatment response based on the sum of the diameters of all target lesions. Main endpoint: OS was determined as the duration from the initiation of first-time immunotherapy until death from any cause or, alternatively, the last date of follow-up.

### 2.5 Statistical analysis

SPSS 25.0 and R software (3.6.1) were used to statistically analyze the clinical data, and GraphPad Prism 8.0 was used for graphing. We used X-tile software 3.6.1 to figure out the optimal cut-off values of SII, PNI, and total points obtained from the nomogram. We performed a Spearman correlation analysis to assess the connection between the PNI and the SII. The relationships between SII‐PNI score and the baseline characteristics were examined by chi‐square test for categorical variables. The study utilized the Kaplan-Meier model to conduct survival analysis. We carried out both univariate analysis and multivariate analysis using the Cox proportional hazards regression model. To assess the relative risks, we examined the hazard ratios (HR) along with their 95% confidence interval (CI). The significant independent risk factors identified in the multivariate analysis were incorporated into the construction of the nomogram. To assess the reliability and predictive accuracy of the nomogram, we performed the ROC curve, calibration plots, and DCA curve. P values<0.05 were considered statistically significant.

## 3 Results

### 3.1 Demographic data of patients

**Figure 1:**
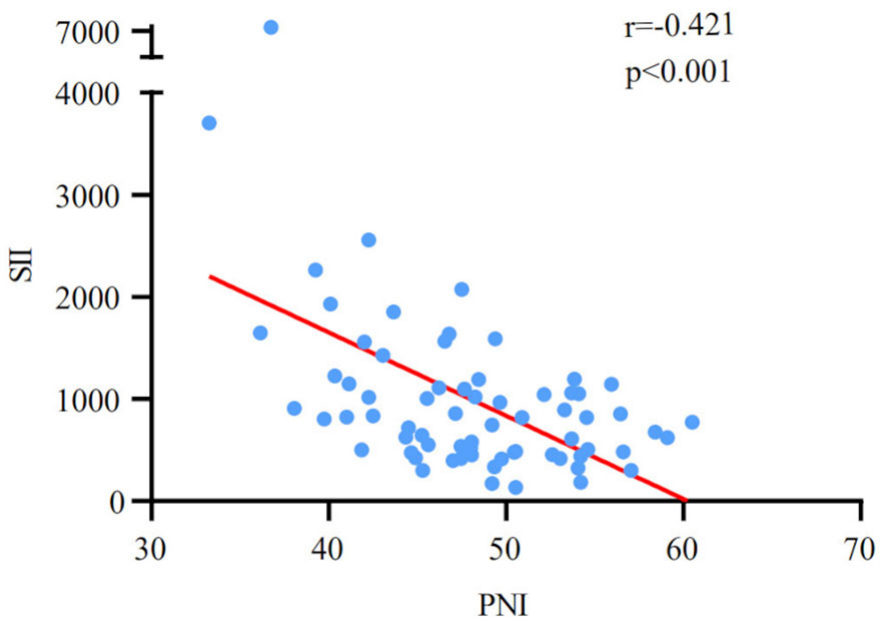
Correlation analysis between pre-treatment SII and PNI.

**Table 1** provides a summary of the demographic data of 70 newly treated ES-SCLC patients engaged in the study. 54 males (77.1%) and 16 females (22.9%) were included. The patients’ median age was 60 years, with a range of 34 to 92. 34 cases (48.6%) had a smoking history. 25 cases received chest radiation therapy (35.7%). 64 cases were treated with Durvalumab (91.4%), and 6 cases were treated with Atezolizumab (8.6%). After four cycles of treatment, the tumor response led to the division of 70 patients into the following categories: PR (48 cases) and non-PR (22 cases). The patients had a median OS of 17.4 months. The median values of the SII and PNI before PD-L1 inhibitors with first-line chemotherapy were 1003.4 (ranging from 131.7 to 7415.2) and 48.0 (ranging from 33.3 to 60.5), respectively. Meanwhile, a significant negative correlation was discovered between SII and PNI (r=-0.421, p<0.001; **Figure 1**).

**Table 1:** Clinical characteristics of the patients.

Characteristics	No (%)
Age(year)	
≤65	50 (71.4)
>65	20 (28.6)
Gender	
female	16 (22.9)
male	54 (77.1)
BMI (Kg/m ²)	
≤24	34 (48.6)
>24	36 (51.4)
ECOG	
0-1	56 (80.0)
2	14 (20.0)
Smoking history	
Never	36 (51.4)
Ever	34 (48.6)
PD-L1 category	
Durvalumab	64 (91.4)
Atezolizumab	6 (8.6)
Chest radiation therapy	
Never	45 (64.3)
Ever	25 (35.7)
Liver metastasis	
No	53 (75.7)
Yes	17 (24.3)
Bone metastasis	
No	60 (85.7)
Yes	10 (14.3)
Brain metastasis	
No	57 (81.4)
Yes	13 (18.6)

### 3.2 Relationships between the pre-treatment SII/PNI and the tumor response and OS

**Figure 2:**
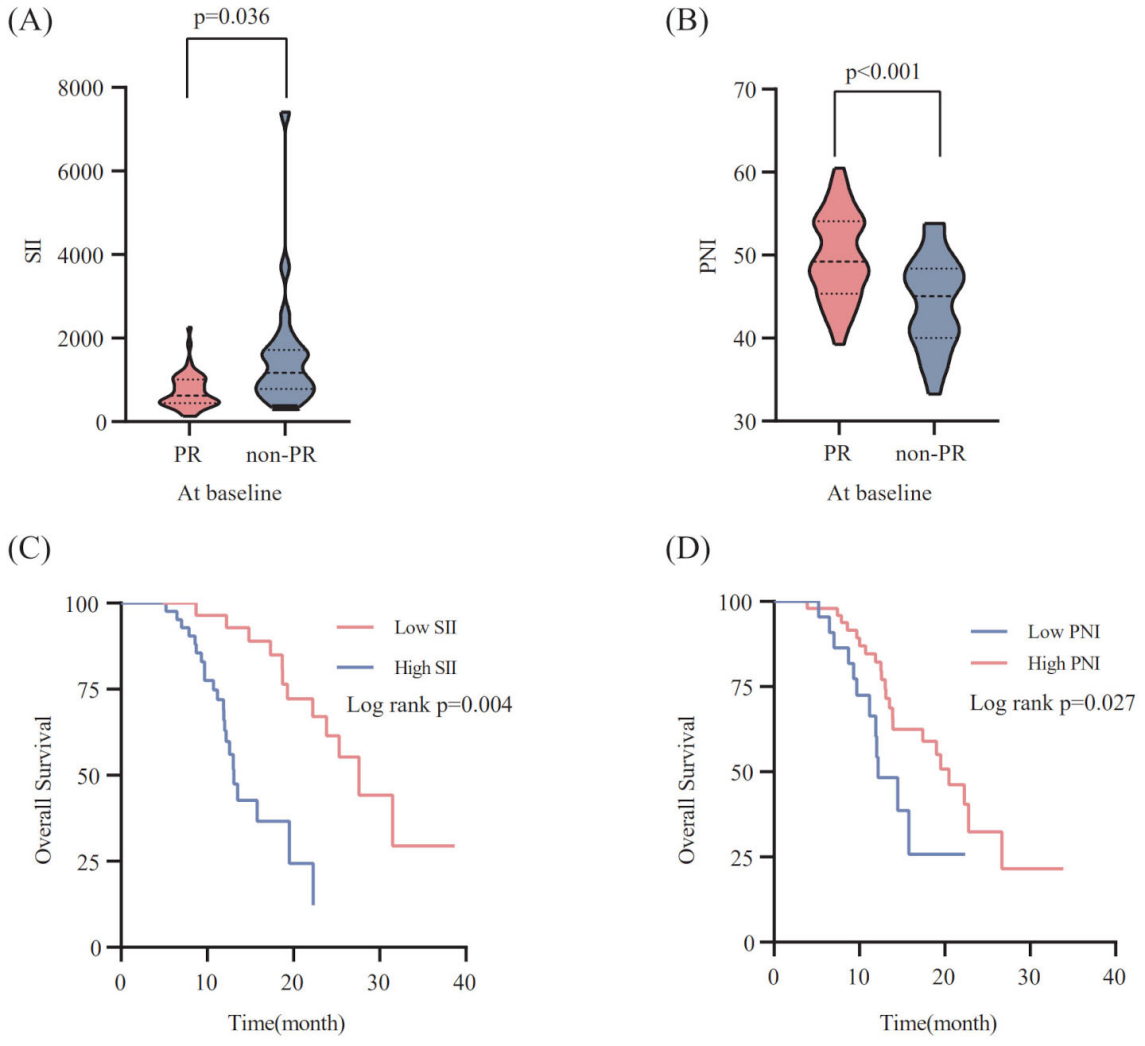
Relationship between the SII **(A)** / PNI **(B)** at baseline and the response to PD-L1 inhibitors combined with chemotherapy. The Kaplan-Meier survival curves for overall survival according to pre-treatment SII **(C)** /PNI **(D)**.

According to X-tile 3.6.1 software, the optimal cut-off values of pre-treatment SII and PNI were 623.91 and 45.25, respectively. The 70 ES-SCLC patients were classified into two groups: low SII (SII ≤ 623.91) and high SII (SII>623.91), with 28 and 42 patients, respectively. Similarly,70 patients were sorted into two groups: low PNI (PNI ≤ 45.25) and high PNI (PNI>45.25), with 48 and 22 patients. In order to examine the relationship between the patient’s systemic inflammation/immune status and curative effect, we next assessed how the baseline SII and PNI correlate with the tumor response. The PR group’s baseline SII was notably lower compared to that observed in the non-PR group(p=0.036) (**Figure 2A**). Meanwhile, the PR group had a significantly higher pre-treatment PNI level than the non-PR group (p<0.001) (**Figure 2B**).

To explore the correlation between the baseline SII/PNI and OS, we analyzed the OS of patients from different levels. Patients with a high SII level (p=0.004; **Figure 2C**) had markedly poorer OS, whereas those with a high PNI level showed improved OS than patients in the low PNI group (p=0.027; **Figure 2D**).

### 3.3 Correlations between the SII-PNI score and the clinicopathological characteristics, and the short-term efficacy

The patients were classified into three groups on the basis of SII and PNI: 0, characterized by both a low SII score and high PNI score (n=25); 1, presented with either a high SII score or a low PNI score (n=26); 2, exhibited a high SII score and a low PNI score (n=19). No notable statistical differences were found in age, gender, smoking history, ECOG PS, BMI, PD-L1 category, and metastasis site (all p>0.05) among the three groups, according to the correlation analysis between SII-PNI score and the baseline clinicopathological characteristics (**Table 2**). There was also no notable association observed between the pre-treatment SII-PNI score and a history of receiving chest radiation therapy (p>0.05).

**Table 2:** The clinicopathological characteristics according to SII-PNI scores.

Characteristics	SII-PNI score			p value
	0(n=25)	1 (n=26)	2 (n=19)	
Age				0.229
≤65	19	20	11	
>65	6	6	8	
Gender				0.876
male	20	19	15	
female	5	7	4	
BMI (Kg/m ²)				0.329
≤24	15	10	9	
>24	10	16	10	
ECOG PS				0.116
0-1	22	21	13	
2	3	5	6	
Smoking history				0.536
Never	12	13	11	
Ever	13	13	8	
PD-L1 category				0.815
Durvalumab	23	23	18	
Atezolizumab	2	3	1	
Chest radiation therapy				0.400
Never	16	14	15	
Ever	9	12	4	
Liver metastasis				0.541
No	22	15	16	
Yes	3	11	3	
Bone metastasis				0.377
No	21	21	18	
Yes	4	5	1	
Brain metastasis				0.426
No	21	22	14	
Yes	4	4	5	
Tumor response				0.004
PR	21	19	8	
Non-PR	4	7	11	

All patients received whole chest and abdominal enhanced CT scans after 4 cycles of PD-L1 inhibitors combined with chemotherapy. A notable difference in the SII-PNI score was observed between the PR group and the non-PR group (**Table 2**). Patients who achieved a PR had a significantly lower SII-PNI score compared to those with non-PR.

### 3.4 Relationship between the SII-PNI score and prognosis

**Figure 3:**
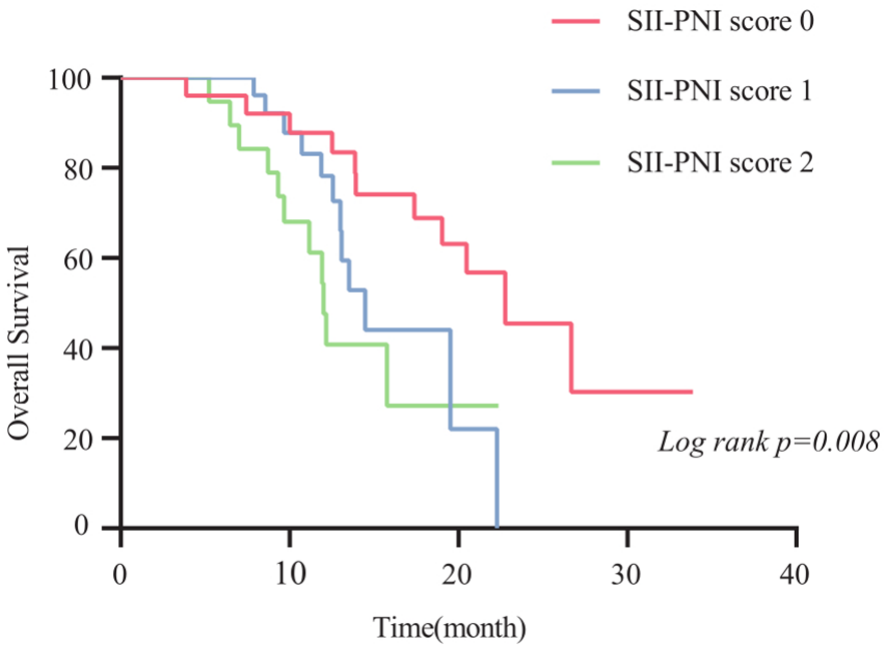
Comparison of overall survival time of patients with different SII-PNI scores.

The 2-year OS rate was 62.9%, with a median OS of 17.4 months (95% CI,11.4-23.4 months). Patients with SII-PNI scores of 0, 1, and 2 had median OS of 22.8, 14.5, and 12.0 months, respectively. Kaplan-Meier survival analysis indicated that the best outcome was observed in the SII-PNI score of 0 group (median OS 22.8 months; 95% 16.5-29.0 months), whereas the worst outcome was observed in the SII-PNI score of 2 group (median OS 12.0 months; 95% 10.8-13.2 months; p=0.008, **Figure 3**). Cox regression analyses were conducted to evaluate OS (**Table 3**). The multivariate analysis indicated that liver metastasis (p=0.025), poorer tumor response (p=0.002), and SII-PNI score (p=0.036) had an independent association with a less favorable OS. We observed a nearly four-fold change in the HR of survival between the SII-PNI score of 2 and the SII-PNI of 0 groups. The hazard ratio (HR) of the SII-PNI score of 1 group was 1.8 times higher than that of the SII-PNI score of 0 group.

**Table 3:** Univariate and multivariate analyses of the clinicopathological characteristics for OS.

Independent factor	Univariate analysis			Multivariate analysis		
	HR	95%CI	p value	HR	95%CI	p value
Age			0.672			
≤65	1.000	Reference				
>65	1.174	0.559-2.467				
Gender			0.22			
female	1.000	Reference				
male	0.613	0.280-1.339				
BMI (Kg/m ²)			0.358			
≤24	1.000	Reference				
>24	1.388	0.690-2.791				
ECOG			0.457			
0-1	1.000	Reference				
2	0.696	0.267-1.810				
Smoking history			0.226			
Never	1.000	Reference				
Ever	0.654	0.329-1.300				
PD-L1			0.174			
Atezolizumab	1.000	Reference				
Durvalumab	0.514	0.197-1.340				
Chest radiation therapy			0.206			
Never	1.000	Reference				
Ever	0.617	0.292-1.304				
Liver metastasis			0.019			0.025
No	1.000	Reference		1.000	Reference	
Yes	2.364	1.155-4.841		2.497	1.124-5.545	
Bone metastasis			0.824			
No	1.000	Reference				
Yes	1.101	0.473-2.560				
Brain metastasis			0.755			
No	1.000	Reference				
Yes	1.143	0.495-2.638				
Tumor response			<0.001			0.002
PR	1.000	Reference		1.000	Reference	
Non-PR	4.477	2.064-9.711		3.688	1.628-8.354	
SII-PNI score			0.010			0.036
0	1.000	Reference		1.000	Reference	
1	2.419	0.987-5.932		1.803	0.687-4.730	
2	4.042	1.608-10.160		3.646	1.359-9.782	

### 3.5 Construction and validation of the prognostic nomogram

**Figure 4:**
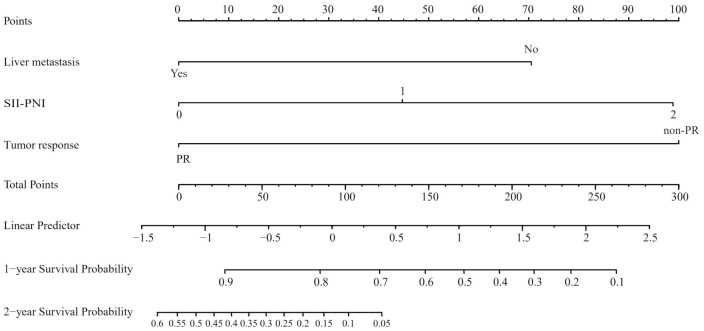
The nomogram model based on liver metastasis, tumor response, and SII-PNI score to predict the probabilities of 1- and 2-year OS.

**Figure 5:**
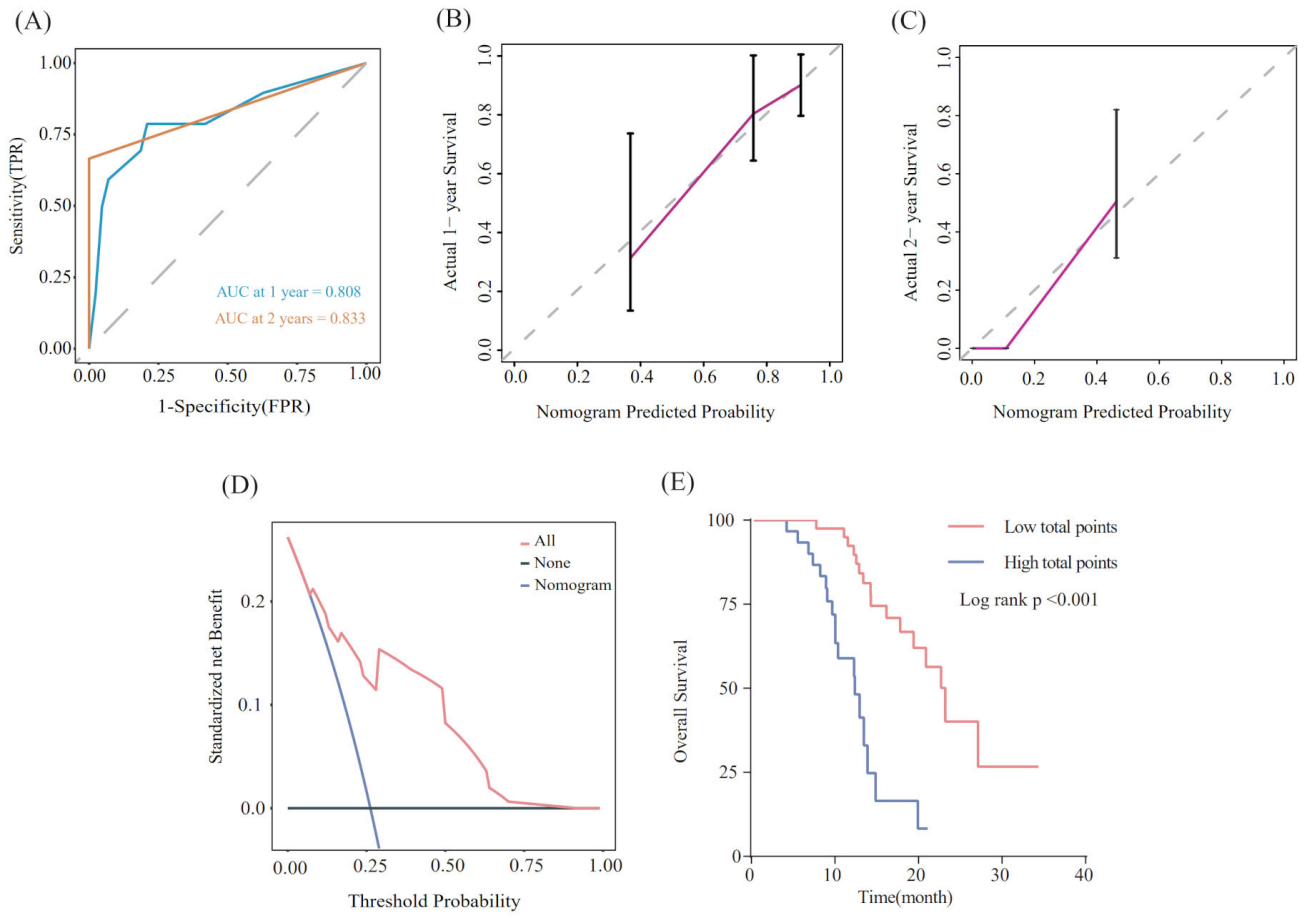
Evaluation of the nomogram model. **(A)** ROC curve that predicts 1- and 2-year survival. **(B, C)** Calibration curves of prediction models for predicting 1- and 2-year survival. **(D)** Decision Curve Analysis. **(E)** Kaplan-Meier survival curves of patients with high and low-risk groups.

Utilizing the findings from both univariate and multivariate analyses, liver metastasis, tumor response, and SII-PNI score were incorporated to develop a nomogram for predicting OS in ES-SCLC patients (**Figure 4**). The AUC values of 1 and 2 year were 0.808 and 0.833, respectively (**Figure 5A**). Besides, the calibration curves (**Figure 5B** **/** **5C**) demonstrated the exceptional prognostic capability of the nomogram. The calibration curves indicate that the model has a good fit between the standard and correction curves. The DCA curve (**Figure 5D**) demonstrated that the nomogram model’s net income peaks when risk thresholds range from 0.1 to 0.75. X-tile software 3.6.1 identified the optimal cut-off value of the total points as 98. Kaplan-Meier analysis was conducted to illustrate the stratification ability of the nomogram. Survival analysis identified significant differences between the groups classified as low-risk and high-risk (**Figure 5E**).

## 4 Discussion

For patients with extensive-stage SCLC, the median survival time typically ranges from 9 to 10 months, and the first-line standard chemotherapy is the main treatment option. The emergence of ICIs has changed the treatment approach of SCLC. ICIs prevent tumor cells from completing immune evasion through immune checkpoints, promoting the immune system to fully exert its anti-tumor activity (18, 19). Chemotherapy can induce immunogenic cell death of tumor cells, improve the ability of immune system to recognize the tumor cells (20), upregulate the expression of PD-L1, and enhance the efficacy of ICIs (21). Therefore, it is believed that chemotherapy and immunotherapy have a synergistic effect. The current research results show that the combination of PD-L1 inhibitors and chemotherapy can benefit patients, but the factors affecting its efficacy and prognosis are still unclear. Immunotherapy can also bring serious adverse reactions, and only a portion of patients can benefit from it. Therefore, before PD-L1 inhibitor combined with first-line chemotherapy is utilized to those suffering from ES-SCLC, it would be advantageous to have a simple indicator that can reliably predict the efficacy and prognosis. This would significantly assist in formulating and selecting individualized treatment regimens.

High expression of PD-L1 is associated with greater immune benefits in NSCLC and some malignant tumors (22–24). However, unlike NSCLC, biopsy samples of SCLC often contain necrotic tissue, so it is usually not possible to provide sufficient samples for PD-L1 detection. In addition, among detectable specimens, compared with NSCLC, the expression frequency of PD-L1 in SCLC is significantly lower (25, 26), with only about 25% of SCLC patients showing positive PD-L1 expression in tumor cells (27). Moreover, due to the strong heterogeneity of SCLC, there are significant differences in PD-L1 detection results, which limits the application of PD-L1 in predicting the efficacy of immunotherapy in SCLC populations (28). TMB has been approved by the FDA as a predictive indicator for the efficacy of pan-tumor immunotherapy (29). However, issues such as insufficient sample size for testing and inconsistent cut-off values also prevent TMB from accurately predicting the efficacy of SCLC immunotherapy. Increasing numbers of investigations have discovered the predictive role of hematological indicators in the efficacy of cancer immunotherapy. Finding effective biomarkers from economically available test items such as blood routine and liver and kidney function is currently one of the most active areas of cancer treatment research.

The occurrence and development of malignancies are heavily influenced by systemic nutrition and inflammation status. An increasing number of studies have demonstrated the correlation between chronic inflammation and an elevated risk of getting cancer (10). A high SII level has shown a strong correlation with poorer prognosis in malignant solid tumors (30–32). Many studies have also identified that the SII correlates with the prognosis of SCLC patients. In addition, the nutritional status also matters greatly in disease progression and prognosis of cancers (8). PNI is a widely used nutritional index that was originally introduced to evaluate patients’ nutritional status before surgery and to predict the risk of complications after surgery (33, 34). More and more studies have found that PNI has prognostic value for many types of cancers, including lung (35), gastric (36), colorectal (37), and hepatocellular carcinoma (38). Previous studies have indicated that the SII-PNI score is a significant prognostic tool in NSCLC (16), gastric cancer (17, 39), and urothelial carcinoma (40). As far as we know, we are pioneering in integrating the SII and PNI of ES-SCLC patients to define the SII-PNI score as a novel indicator for predicting efficacy and prognosis.

Tumor response plays a crucial role in predicting the survival outcome of ES-SCLC patients treated with PD-L1 inhibitors. It is challenging to anticipate how tumors will respond based solely on clinical pathological data before immunotherapy. We pay attention to SII and PNI to address the difficulties in predicting tumor response. The results of this study indicated a significant correlation between the baseline SII-PNI score and the tumor response to immunotherapy. Patients with lower SII-PNI scores were found to have a higher possibility of responding well to PD-L1 inhibitors. These findings indicated that the SII-PNI score may function as a potential biomarker for predicting short-term efficacy in ES-SCLC patients taking immunotherapy with chemotherapy.

The prognostic analysis revealed that a higher SII-PNI score has a strong association with a poorer prognosis. The following elements could potentially contribute to the SII-PNI score’s predictive capability: (1) An elevated SII-PNI score suggests that the count of neutrophils or platelets has increased proportionally compared to the lymphocyte count present within the blood sample. Elevated neutrophil count has been seen having as an unwelcome role in tumor development, invasion, metastasis, and resistance to treatment (41). Previous studies have shown that neutrophils can suppress anti-tumor cytotoxic T lymphocytes *in vitro* and produce cytokines that promote angiogenesis (42). Platelets can activate a large number of bioactive factors, increase tumor vascular permeability, and serve as a shield for tumor cells against the infiltration of cytotoxic lymphocytes and natural killer cells, as well as promote the development of an immunosuppressive tumor microenvironment (43). (2) An elevated SII-PNI score also reflects a relative reduction in lymphocyte levels. As immune cells in the body, lymphocytes can inhibit cancer cell division and metastasis by inducing cell apoptosis, playing a role in immune monitoring and tumor defense (44, 45). The decrease in lymphocyte count can also reduce the efficacy of ICIs, which primarily release inhibitory signaling properties of T lymphocytes. Research has shown that increased infiltration of tumor-infiltrating lymphocytes (TILs) is associated with better efficacy and prognosis of immunotherapy in patients with solid tumors (46). (3) Malnutrition and inflammatory status of the body can both affect the synthesis of serum albumin. Low levels of albumin often suggest malnutrition and weakened immune function in patients. It can also reflect the body’s inflammatory status and anti-tumor immune response. Cachexia can also lead to disease progression (47).

We found liver metastasis, tumor response, and SII-PNI score were independently associated with OS. Following this, we developed a nomogram using the above-mentioned factors. To ensure our results were reliable and accurate, we conducted additional analyses using ROC curves and calibration curves. These statistical tools confirmed that our model had excellent predictive accuracy, with its prediction closely aligning with actual observations. In addition, we implemented a system stratifying mortality risk that categorizes patients into high- and low-risk subgroups. This system facilitates the effective management of risk stratification and the provision of individual treatment. A significant difference in OS was identified between the two different levels of risk.

Nevertheless, this study had certain limitations. Because PD-L1 inhibitors are prohibitively expensive, only a small population of patients have access to them. This study was constrained by a limited sample size. Additionally, there was a lack of an external validation cohort to further validate the effectiveness and predictive power of the nomogram for patients with ES-SCLC. Due to the small sample size of the study and the overfitting issues in the model, the SII-PNI score system has a certain risk of bias. Thirdly, systemic inflammation and nutritional status can be affected by various factors unrelated to cancer. However, this study did not address the potential confounding factors that may affect SII-PNI scores, such as concurrent infections or drug use (such as antibiotics or proton pump receptor inhibitors). Lastly, due to the fact that most patients have not undergone PD-L1 expression level and TMB testing, the correlation between these indicators and efficacy has not been explored. In the future, we may consider the possibility of combining the SII-PNI score with biomarkers such as PD-L1 expression level and TMB to improve the prognostic accuracy. In order to explore predictive biomarkers for ES-SCLC immunotherapy and identify the most suitable population for immunotherapy, more large-scale, prospective, and multi-center studies are still needed.

## 5 Conclusion

In summary, our study showed that the SII-PNI score can effectively predict the efficacy and prognosis of extensive-stage SCLC patients treated with PD-L1 inhibitors combined with first-line chemotherapy. Baseline hematological inflammation indicators can be obtained through blood routine, which has the advantages of simplicity, economy, and minimal trauma. Clinicians should take into account this innovative biomarker when making clinical decisions, managing risk stratification, and selecting the best patients for PD-L1 inhibitors.

## Data Availability

The raw data supporting the conclusions of this article will be made available by the authors, without undue reservation.
